# Vitamin A family suppresses periodontitis by restoring mitochondrial metabolic reprogramming in macrophages through JAK-STAT pathway

**DOI:** 10.3389/fgene.2025.1505933

**Published:** 2025-01-28

**Authors:** Zishuo Cheng, Shun Huang, Qiya Tang, Danlan Zhang, Lan Huang

**Affiliations:** Chongqing Key Laboratory for Oral Diseases and Biomedical Sciences, Chongqing Municipal Key Laboratory for Oral Biomedical Engineering of Higher Education, Stomatological Hospital of Chongqing Medical University, Chongqing, China

**Keywords:** metabolic reprogramming, JAK-STAT pathway, vitamin A, inflammation, mitochondiral

## Abstract

**Objective:**

Mitochondrial metabolic reprogramming in macrophages is crucial in the development and progression of inflammation. Given vitamin A’s antioxidant properties and its therapeutic effects on inflammation, this study aims to elucidate how vitamin A influences mitochondrial metabolic reprogramming in inflammatory states, specifically in periodontitis, through genetic bioinformatics and experimental methods.

**Method:**

The study utilized the GSE16134 dataset from the Gene Expression Omnibus (GEO) database, focusing on human periodontitis. Vitamin A-targeted genes (ATGs) were identified and analyzed using CIBERSORT to explore their role in inflammation. Cluster analysis revealed two phenotypes associated with ATGs, showing differential expression of genes like *COX1, IL-1β*, and *STAT3*, and immune activation patterns. Weighted Gene Co-expression Network Analysis (WGCNA) identified 145 markers correlated with ATG-guided phenotypes and inflammation. Machine learning models, combined with Gene Set Variation Analysis (GSVA), identified five key genes (*RGS1, ACAT2, KDR, TUBB2A, TDO2*) linked to periodontitis. Cell Type-Specific Enrichment Analysis (CSEA) highlighted macrophages as critical in metabolic reprogramming, validated by external datasets with an AUC of 0.856 in GSE10334 and 0.750 in GSE1730678. Experimental validation showed vitamin A’s role in suppressing endoplasmic reticulum stress and altering mitochondrial dynamics, as well as metabolic reprogramming influencing inflammation via the STAT3 pathway in RAW 264.7 cells.

**Results:**

The study identified 13 differentially expressed ATGs in periodontitis, showing strong correlations with inflammation, particularly in plasma cells, macrophages, dendritic cells, neutrophils, and mast cells. Two ATG-guided phenotypes were identified, differing in gene expression and immune activation. WGCNA and machine learning models identified 145 markers and five key genes associated with periodontitis. GSVA and CSEA analyses highlighted the JAK-STAT pathway and macrophage involvement in metabolic reprogramming. Experimental data confirmed vitamin A’s effects on mitochondrial dynamics and metabolic reprogramming through the STAT3 pathway.

**Conclusion:**

The study demonstrates that vitamin A’s therapeutic effect on periodontitis is mediated through JAK-STAT pathway-guided mitochondrial metabolic reprogramming in macrophages. It identifies two genetic and immune-related phenotypes and five genetic identifiers associated with periodontitis risk.

## 1 Introduction

Periodontitis (PD) is a common oral disease primarily caused by a chronic infection of the gums and surrounding tissues. It usually begins as gingivitis, characterized by gum redness, swelling, and bleeding. Left untreated, it can progress to periodontitis, leading to gum recession, alveolar bone loss, and eventually, tooth mobility and loss (Slots, 2017). The main causative factors of periodontitis are dental plaque and calculus, but genetics, smoking, and poor oral hygiene habits can also increase the risk. Early-stage periodontitis can be controlled through improved oral hygiene and professional treatment, while severe cases may require surgical intervention (Teles et al., 2022). Regular dental check-ups and cleanings are essential for preventing periodontitis.

Periodontitis, despite being manageable through mechanical treatments such as scaling and root planning, remains a chronic condition with a significant risk of recurrence. This persistent threat is closely linked to the concept of inflammation-mediated metabolic reprogramming (Bartold and Van Dyke, 2017). When the body encounters a prolonged inflammatory response, as observed in periodontitis, it triggers complex biochemical pathways that lead to alterations in cellular metabolism (O’Neill et al., 2016). These changes, which can occur at both the local periodontal tissues and systemic levels, create an environment conducive to the reactivation of the disease even after clinical signs have been alleviated through treatment (Jha et al., 2015). One key aspect of this metabolic reprogramming is the shift in energy production within immune cells.

In response to chronic inflammation, immune cells, such as macrophages, undergo a transition from oxidative phosphorylation to glycolysis, a process known as the Warburg effect (Kelly and O’Neill, 2015). This shift not only supports the immediate energy needs required for sustaining the inflammatory response but also results to the production of pro-inflammatory mediators that perpetuate the inflammation. As a result, even after mechanical removal of plaque and calculus, the inflammatory processes may persist, leading to further tissue damage and disease recurrence (Viola et al., 2019). Moreover, this metabolic reprogramming can also influence the regenerative capacity of the periodontal tissues. The altered metabolic state of fibroblasts and other resident cells within the periodontal ligament may impair their ability to repair and regenerate the damaged tissues effectively (Zebrowitz et al., 2022). This compromised regenerative potential contributes to the long-term challenges in fully resolving periodontitis.

Based on evidence-based research, vitamins have demonstrated a potential role in reducing the risk of periodontitis and improving its prognosis (Gutierrez Gossweiler and Martinez-Mier, 2020). Vitamins such as vitamin C, D, and E are known to possess antioxidant and anti-inflammatory properties, which are thought to help mitigate the inflammatory processes involved in the development of periodontal disease (Sulijaya et al., 2019). Recent meta-analysis has shown that high-dose vitamin A intake is negatively correlated with the likelihood of developing periodontal disease, highlighting the important role of vitamin A in preventing periodontal disease. One of the studies included 45 effect sizes from 23 observational studies, with a total of 74,488 participants (Mi et al., 2024). The results indicated that higher levels of vitamin A intake are negatively associated with the prevalence of periodontal disease. Despite the promising role of vitamins in managing periodontal health (Gutierrez Gossweiler and Martinez-Mier, 2020), there is a noticeable gap in the research concerning the specific interaction between periodontitis and vitamin A through inflammation and metabolic reprogramming. While vitamins such as C and D have been more extensively studied for their roles in antioxidant defense and immune modulation (Hu et al., 2022), the potential involvement of vitamin A in these processes remains underexplored in the context of periodontitis. Vitamin A is known for its role in maintaining epithelial integrity and modulating immune responses, yet its specific impact on the metabolic pathways altered by chronic periodontal inflammation has not been thoroughly investigated (Bar-El Dadon and Reifen, 2017). Recent experimental studies highlight that vitamin A could potentially affect inflammatory processes and metabolic reprogramming (Tejón et al., 2015). For example, α-ketoglutarate can modulate the metabolism of T cells and macrophages, while retinoic acid (RA) can suppress inflammatory responses and promote the differentiation of immune cells toward an anti-inflammatory phenotype by altering intracellular metabolic pathways (Erkelens and Mebius, 2017). Additionally, the metabolic products of vitamin A, such as retinoic acid, can act on metabolic sensors like peroxisome proliferator-activated receptor gamma (PPAR-γ), which plays a crucial role in regulating cellular metabolism and inflammatory responses. By activating these receptors, vitamin A can inhibit the expression of inflammatory genes and promote metabolic reprogramming (Czarnewski et al., 2017; Takeda et al., 2016). Furthermore, the metabolic products of vitamin A can affect mitochondrial function by altering the mitochondrial respiratory chain and energy metabolism pathways, thereby modulating inflammatory responses. For instance, retinoic acid can promote mitochondrial biogenesis and improve mitochondrial function, leading to a reduction in the production of reactive oxygen species (ROS) in inflammatory cells (Mills et al., 2016; Liu et al., 2017). It is crucial to understand how vitamin A might interact with inflammatory mediators and metabolic pathways that are reprogrammed in response to chronic periodontitis (Dommisch et al., 2018). Such knowledge could provide deeper insights into developing targeted therapeutic strategies that incorporate vitamin A or its derivatives to manage periodontitis more effectively. Still, these findings are primarily speculative and require further empirical validation.

This study was dedicated to elucidating the mechanisms through which vitamin A influences inflammation and metabolic reprogramming in periodontal tissues, Involving exploring its role in regulating the immune response, affecting cellular metabolism, and its potential to enhance or suppress inflammatory pathways. Understanding these interactions could pave the way for innovative therapeutic approaches that integrate nutritional interventions with traditional periodontal therapies, potentially offering a more comprehensive management strategy for periodontitis.

## 2 Material and methods

### 2.1 Data processing

The datasets utilized in this study were sourced from the GEO database, accessible at https://www.ncbi.nlm.nih.gov/geo/for further details. Specifically, dataset GSE16134, based on the GPL570-9,606 platform, was selected for analysis. This dataset comprises 310 gingival papillae samples collected from 120 subjects undergoing periodontal surgery, of which 241 were classified as “diseased” and 69 as “healthy.” The GSE10334 dataset, also based on the Affymetrix Human Genome U133 Plus 2.0 Array (GPL570-9606) platform, includes 247 gingival papillae samples from 90 periodontitis patients, with 183 classified as “diseased” and 64 as “healthy.” This dataset was used to confirm the diagnostic efficacy of disease related gene-based signatures. The GSE173078 dataset was taken from the gingival tissues of 12 periodontitis patients and 12 normal individuals, based on Illumina HiSeq 4000 (*Homo sapiens*, GPL20301).

For dataset GSE16134 and 10334, tissue samples were obtained from patients diagnosed with moderate to severe periodontitis undergoing periodontal surgery (Tonetti et al., 2018). The diagnostic criteria were as follows: “Diseased” sites exhibited bleeding on probing (BoP), had an interproximal probing depth (PD) of ≥4 mm, and concomitant attachment loss (AL) of ≥3 mm. In contrast, from the same population,“Healthy” sites displayed no BoP, had a PD of ≤4 mm, and AL of ≤2 mm. For GSE173078, the periodontitis samples were collected from sites with PD ≥ 5 mm, CAL ≥3 mm, radiographic bone loss beyond the coronal third of the root, and bleeding on probing (BoP). The periodontally healthy samples were collected from sites with PD ≤ 3 mm, no CAL, and no BoP (Kim et al., 2021). A total of 13 vitamin A targeted genes (ATGs) were extracted from the original research on vitamin A, and further investigations were conducted based on these genes (Ye et al., 2022).

### 2.2 Assessment of immune cell infiltration and correlation analysis between ATGs and infiltrated immune cells

The CIBERSORT algorithm (https://cibersort.stanford.edu/) along with the LM22 signature matrix was utilized to estimate the relative abundances of 22 immune cell types in each sample, based on the analyzed gene expression data. CIBERSORT employs Monte Carlo sampling to calculate an inverse fold product p-value for each sample. Only samples with p-values below 0.05 were deemed to have accurate immune cell fraction estimates. The sum of the proportions of the 22 immune cell types in each sample equaled 1 (Newman et al., 2015).

To further investigate the relationship between ATGs and immune cell properties related to periodontitis (PD), we analyzed the correlation coefficients between ATG expression levels and the relative proportions of immune cells. Spearman’s correlation coefficient was employed, and correlations were considered statistically significant if the p-value was below 0.05. The findings were then visualized using the “corrplot” R package (version 0.92).

### 2.3 Unsupervised clustering of PD patients

Initially, 13 vitamin A ATGs were identified based on prior studies (Tsvetkov et al., 2022). Utilizing the expression profiles of these 13 ATGs, we conducted unsupervised clustering analysis using the “ConsensusClusterPlus” R package (version 2.60) (Wilkerson and Hayes, 2010). The k-means algorithm was employed with 1,000 iterations to classify periodontitis (PD) samples into distinct clusters. A maximum number of subtypes (k = 2) was selected, and the optimal number of clusters was determined through a comprehensive evaluation of the cumulative distribution function (CDF) curve, consensus matrix, and a consistent cluster score greater than 0.9. The parameters used for the analysis included a resampling rate of 80%, 100 resampling iterations, and a cluster proportion of 0.8.

### 2.4 Gene set variation analysis (GSVA) analysis

GSVA enrichment analysis was performed to identify differences in enriched gene sets between distinct ATGs clusters using the “GSVA” R package (version 2.11). The files “c2. cp.kegg.v7.4. symbols” and “c5. go.bp.v7.5.1. symbols” were sourced from the MSigDB database to support the GSVA analysis. To detect differentially expressed pathways and biological functions, the “limma” R package (version 3.52.1) was applied, comparing GSVA scores across the various ATGs clusters. A |t value of GSVA score| greater than 2 was considered indicative of significant alterations.

### 2.5 Weighted gene co-expression network analysis (WGCNA)

To identify co-expression modules, Weighted Gene Co-expression Network Analysis (WGCNA) was conducted using the “WGCNA” R package (version 1.70.3) (Langfelder and Horvath, 2008). The top 25% of genes with the highest variance were selected for subsequent WGCNA analysis to ensure robust results. An optimal soft threshold power was chosen to construct a weighted adjacency matrix, which was then converted into a topological overlap matrix (TOM). Modules were identified using the TOMs dissimilarity measure (1-TOM) with a hierarchical clustering tree algorithm, and a minimum module size of 100 was set. Each module was randomly assigned a color. The module eigengene represented the overall gene expression profile for each module. The association between modules and disease status was evaluated using module significance (MS), while gene significance (GS) was defined as the correlation between individual genes and clinical phenotypes.

### 2.6 Construction of predictive model based on multiple machine learning methods

To construct machine learning models based on two distinct ATGs clusters, the “caret” R package (version 6.0.91) was employed to develop a range of models, including a random forest model (RF), support vector machine model (SVM), generalized linear model (GLM), and eXtreme Gradient Boosting (XGB). The RF model, an ensemble learning method, utilizes multiple independent decision trees to predict classification or regression outcomes (Rigatti, 2017). The SVM algorithm generates a hyperplane in the feature space that maximizes the margin to separate positive and negative instances (Gold and Sollich, 2003). The GLM, an extension of multiple linear regression models, provides flexibility in evaluating relationships between normally distributed dependent variables and categorical or continuous independent variables (Nelder and Wedderburn, 1972). The XGB model, which is based on gradient boosting, integrates multiple boosted trees and balances between classification error and model complexity (Chen, 2015).

In this study, distinct clusters were treated as the response variable, while cluster-specific ATGs were selected as explanatory variables. The PD samples were randomly divided into a training set (70%) and a validation set (30%). Model parameters were automatically optimized using grid search via the “caret” package, with all models executed using default settings and assessed through 5-fold cross-validation. The “DALEX” package (version 2.4.0) was used to interpret the four machine learning models, as well as to visualize residual distributions and feature importance. The “pROC” R package (version 1.18.0) was utilized to plot the area under the receiver operating characteristic (ROC) curves. Based on these analyses, the optimal machine learning model was identified, and the five most important variables were considered as key predictive genes related to PD. To further validate the diagnostic utility of the model, ROC curve analyses were performed using the GSE173078, GSE10334 and GSE16134 datasets.

Additionally, a nomogram model was developed to assess the occurrence of PD clusters using the “rms” R package (version 6.2.0). Each predictor was assigned a specific score, with the “total score” representing the cumulative sum of these scores. The calibration curve and decision curve analysis (DCA) were applied to evaluate the predictive accuracy of the nomogram model.

### 2.7 Molecular docking

3D-structures of ligand molecules retinol and static were obtained from the PubChem database (https://pubchem.ncbi.nlm.nih.gov/), and the protein STAT3 structure used in this study is PDB ID 6NJS, downloaded from the RCSB Protein Data Bank (www.rcsb.org/). The docking procedure was conducted using CB-DOCK2(https://cadd.labshare.cn/cb-dock2/index.php), a convolutional neural network-based docking tool designed to predict the binding mode and affinity between ligands and target proteins (Liu et al., 2022).

Initially, the 3D structure of the protein STAT3 (PDB ID 6NJS) was processed by CB-DOCK2 to identify potential binding pockets on the protein surface. The tool employs a convolutional neural network to analyze the protein structure and automatically predict possible binding sites, generating scores for each identified pocket based on their likelihood of accommodating the ligand.

Following the identification of binding pockets, the ligand molecules from PubChem were docked into these predicted pockets using the AutoDock Vina algorithm integrated within CB-DOCK2.

### 2.8 Molecular dynamics

AmberTools22 was used for molecular docking studies, obtained from the official AMBER website (https://ambermd.org). The ligand molecules were retrieved from the PubChem database, and the target protein structure was downloaded from the RCSB Protein Data Bank (PDB ID: 6NJS).

The docking process began by preparing the protein structure. Hydrogen atoms were added, and missing residues were reconstructed using LEaP, a tool from the AmberTools suite. The protein was then parameterized using the ff14SB force field. The ligand molecule was prepared by assigning atomic charges using the AM1-BCC method, and parameterization was conducted using the GAFF (General Amber Force Field).

Once the ligand and protein structures were prepared, molecular docking was performed using the SANDER module in Amber, which was employed for energy minimization and conformation sampling. The ligand was placed in the binding site of the protein based on initial predictions by molecular docking. A two-step energy minimization was performed: the first with constraints on the protein backbone, and the second without any constraints to allow for full relaxation of the system.

The binding poses were evaluated by calculating the binding free energies using both the Molecular Mechanics/Generalized Born Surface Area (MM/GBSA) and Molecular Mechanics/Poisson-Boltzmann Surface Area (MM/PBSA) methods.

### 2.9 CCK-8 cell viability assay

To assess the cytotoxicity of VA on RAW264.7 cells, a CCK-8 (Cell Counting Kit-8) assay was performed. RAW264.7 cells were seeded in 96-well plates at a density of 1 × 10^4^ cells per well and allowed to adhere overnight. VA was dissolved in dimethyl sulfoxide (DMSO) and added to the wells at varying concentrations. Cells treated with DMSO alone served as a control. After 24 h of treatment, 10 μL of CCK-8 solution was added to each well and incubated for 2 h at 37°C. Absorbance was measured at 450 nm using a microplate reader to evaluate cell viability. All assays were performed in triplicate, and results are expressed as mean ± SD.

### 2.10 Quantitative real-time polymerase chain reaction (qPCR)

Quantitative real-time polymerase chain reaction (qPCR) was performed using the Applied Biosystems 7,500 Real-Time PCR System with SYBR Green dye to detect the amplification products. Each 20 μL reaction mixture contained 1X SYBR Premix Ex Taq™ II (Takara), 0.3 μM forward and reverse primers (Takara), and 10 ng of cDNA template. Synthetic primer sequences can be found in supplementary material (Supplementary Table S1). All reactions were carried out in 96-well plates, and no-template controls (NTC) were included to check for potential contamination.

The qPCR thermal cycling conditions were as follows: initial denaturation at 95°C for 2 min, followed by 40 cycles of denaturation at 95°C for 15 s and annealing/extension at 60°C for 1 min. After the completion of each reaction, a melt curve analysis was performed to verify the specificity of the amplification. The melt curve was obtained by gradually increasing the temperature from 60°C to 95°C while monitoring the SYBR Green fluorescence signal, ensuring the production of a single, specific amplification product.

Quantification was based on CT values, and the relative expression of the target genes was calculated using the ΔΔCT method, with GAPDH as the reference gene. The experiment was repeated three times, with technical replicates for each run to ensure data reliability.

### 2.11 Detection of reactive oxygen species (ROS) by DCFH fluorescence staining

To measure intracellular ROS levels, RAW264.7 cells were seeded on glass coverslips in 24-well plates and treated with PBS, LPS, or LPS + VA for 24 h. After treatment, cells were incubated with 10 μM of DCFH-DA (2′,7′-dichlorodihydrofluorescein diacetate) for 30 min at 37°C in the dark. Following incubation, cells were washed with PBS and fluorescence images were captured using a fluorescence microscope. The fluorescence intensity was proportional to the ROS content in the cells. The scale bar in the images represents 400 µm. All experiments were performed in triplicate, and data are expressed as mean ± SD.

### 2.12 Calcium content detection by Fluo-4 AM fluorescence staining

The intracellular calcium content was assessed using Fluo-4 AM dye. RAW264.7 cells were plated on glass coverslips in 24-well plates and treated with PBS, LPS, or LPS + VA for 24 h. After treatment, cells were incubated with 5 μM of Fluo-4 AM for 30 min at 37°C in the dark. After incubation, cells were washed with PBS, and fluorescence images were obtained using a fluorescence microscope. The scale bar represents 200 µm. Fluorescence intensity was used to estimate intracellular calcium levels. All experiments were performed in triplicate, with data presented as mean ± SD.

### 2.13 Detection of mPTP opening by Calcein-AM Loading/CoCl₂ quenching assay

Mitochondrial permeability transition pore (mPTP) opening was detected using a Calcein-AM loading/CoCl₂ quenching assay. RAW264.7 cells were seeded in 24-well plates on glass coverslips and treated with PBS, LPS, or LPS + VA for 24 h. Cells were then incubated with 1 μM of Calcein-AM for 30 min at 37°C, followed by treatment with 1 mM CoCl₂ to quench the cytoplasmic calcein fluorescence. Fluorescence images were captured using a fluorescence microscope, and the mitochondrial calcein signal was used to estimate mPTP opening. The scale bar represents 200 µm. All experiments were performed in triplicate, and results are expressed as mean ± SD.

### 2.14 Mito-SOX fluorescence staining to detect mitochondrial reactive oxygen species (mROS)

To measure the level of ROS in mitochondria, RAW264.7 cells were seeded on glass coverslips in 24-well plates and treated with PBS, LPS, or LPS + VA for 24 h. After treatment, the cells were incubated with 5 µM Mito-SOX for 30 min at 37°C in the dark. After incubation, the cells were washed with PBS, and fluorescence images were captured using a fluorescence microscope. The fluorescence intensity is proportional to the mROS content in the cells. The scale bar in the image represents 200 µm. All experiments were performed in triplicate and the data are presented as mean ± SD.

### 2.15 Mitochondrial oxidative respiratory chain (OCR) assay

RAW264.7 macrophages were cultured in DMEM supplemented with 10% fetal bovine serum (FBS) and 1% penicillin-streptomycin at 37°C in a humidified atmosphere containing 5% CO_2_. For the OCR assay, cells were seeded into XF96 cell culture microplates at a density of 1 × 10^4^ cells per well and incubated overnight to allow cell adhesion. The cells were then treated with one of the following conditions: control (PBS), lipopolysaccharide (LPS, 1 μg/mL), LPS combined with vitamin A (LPS + VA), STAT3 inhibitor Stattic, which specifically blocks STAT3 activation by preventing its binding to phosphopeptides, LPS + Stattic, or LPS + Stattic + 20 μM VA. After 24 h of treatment, the medium was replaced with assay medium (non-buffered DMEM), and cells were incubated for 1 h at 37°C in a CO_2_-free incubator before the OCR measurements. Basal respiration was continuously monitored for 1 h.

## 3 Results

### 3.1 Dysregulation of vitamin A regulated targets and activation of the immune responses in PD patients

**FIGURE 1:**
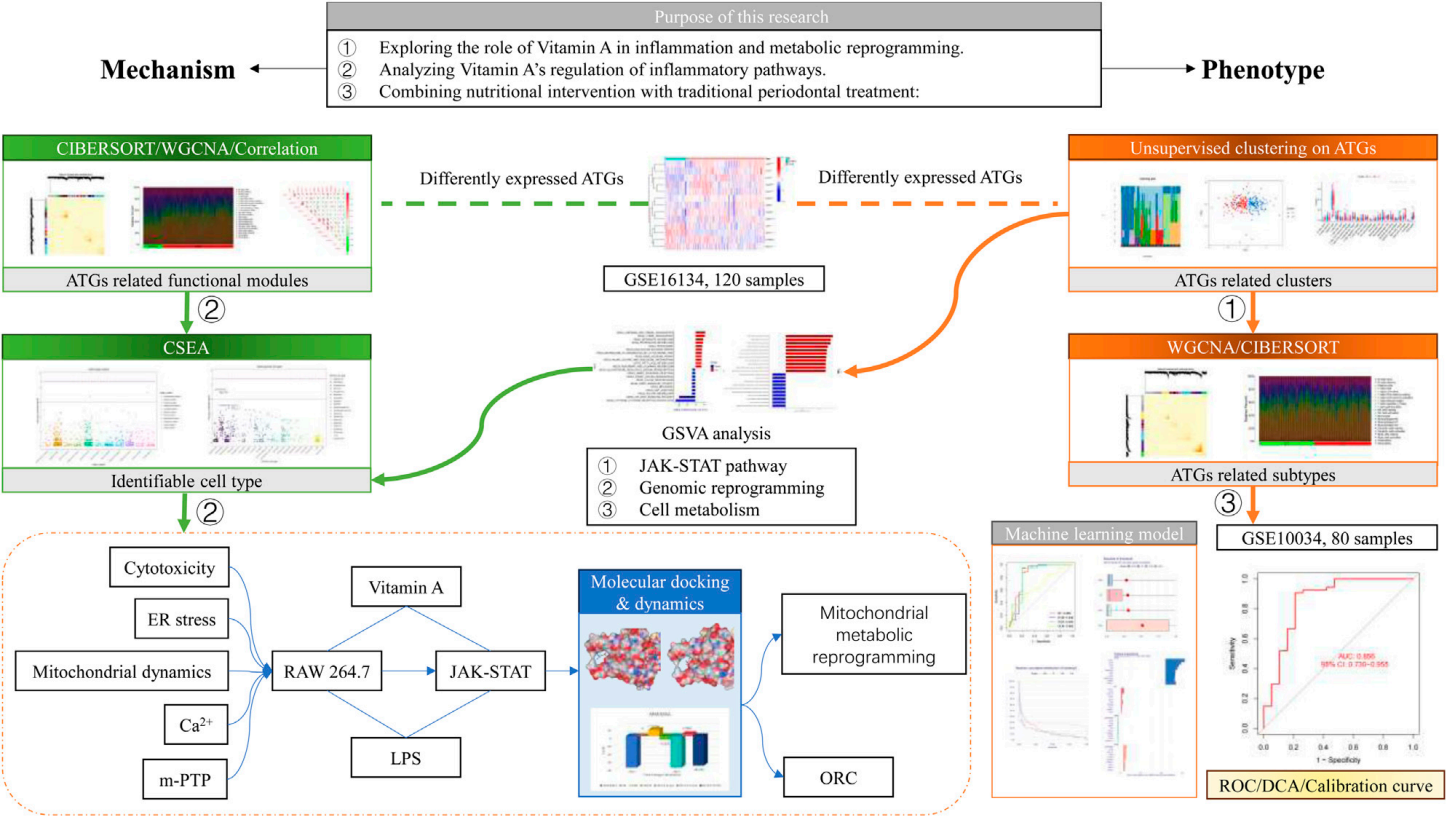
Schematic diagram.

**FIGURE 2:**
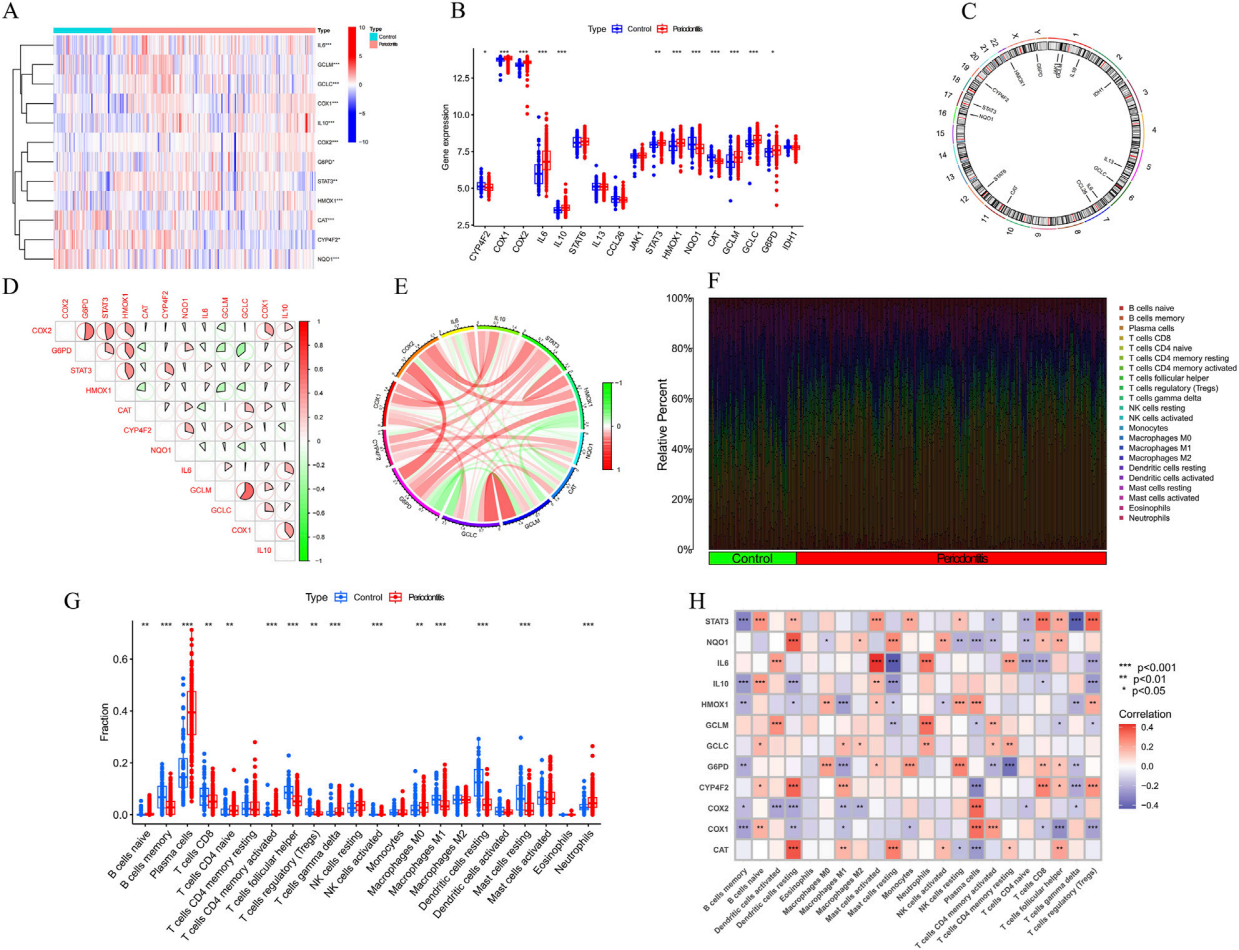
Identification of dysregulation of vitamin A regulated targets and activation of the immune responses in PD patients. **(A–C)** Expression of vitamin A-related genes (ATGs) in periodontitis tissues. Specifically, COX1, COX2, IL10, STAT3, GCLM, GCLC, G6PD, and HMOX1 were upregulated in periodontitis, while NQO1 and CAT were significantly downregulated. **(D, E)** Gene interaction network of differentially expressed ATGs and synergistic relationship. **(F)** Immune infiltration analysis, using the CIBERSORT algorithm, compared the proportions of 22 immune cell types between periodontitis patients and controls. **(G)** specific changes in the immune system of periodontitis patients. **(H)** Correlation analysis revealed a negative relationship between the polarization from M0 to M1 macrophages and the expression levels of G6PD, IL-1β, and HMOX1.

To rigorously investigate the biological roles of vitamin A-regulated targets in the onset and progression of periodontitis (PD), we conducted a systematic analysis of the expression profiles of 31 vitamin A targeting genes (ATGs) using the GSE16134 dataset, comparing PD patients with non-PD controls. The study’s methodology is comprehensively outlined (Figure 1). Our analysis identified these 13 ATGs as differentially expressed genes associated with vitamin A. Specifically, we observed that the expression levels of *COX1*, *COX2*, *IL10*, *STAT3*, *GCLM*, *GCLC*, *G6PD*, and *HMOX1* were elevated in PD tissues, while NQO1, CAT, and ARG1 exhibited significantly lower expression levels relative to non-PD controls (Figures 2A–C). To further explore the implications of these differentially expressed ATGs in PD progression, we performed a correlation analysis. The results revealed a synergistic interaction between the JAK-STAT pathway component STAT3 and the metabolic regulator *G6PD*, whereas an antagonistic relationship was noted between *GCLC* and *G6PD*. Additionally, a gene relationship network diagram provided further insights into the interconnections among these differentially expressed ATGs (Figure 2E). These findings suggest a potential involvement of these genes in the molecular mechanisms underlying PD, warranting further investigation into their specific roles and clinical relevance in PD pathology.

To objectively assess potential differences in the immune system between PD patients and non-PD controls, an immune infiltration analysis was conducted using the CIBERSORT algorithm to compare the proportions of 22 infiltrated immune cell types between the two groups (Figure 2F). The analysis indicated that PD patients exhibited significantly higher infiltration levels of plasma cells and neutrophils (Figure 2G), along with notably lower infiltration levels of memory B cells and follicular helper T cells. These findings suggest that alterations in the immune system may play a role in the pathogenesis of PD. Additionally, the analysis revealed a surprising pattern in macrophage populations, with PD patients showing significantly higher levels of M0 macrophages and lower levels of M1 macrophages. Correlation analysis further demonstrated that the polarization of macrophages from the M0 to M1 type was negatively correlated with the expression levels of *G6PD*, *IL-1β*, and *HMOX1* (Figure 2H). These results imply that vitamin A targeting genes may be crucial factors in modulating the molecular and immune infiltration characteristics observed in PD patients, warranting further investigation into their potential role in PD pathology.

### 3.2 Identification of clusters in PD, gene modules screening and co-expression network construction

**FIGURE 3:**
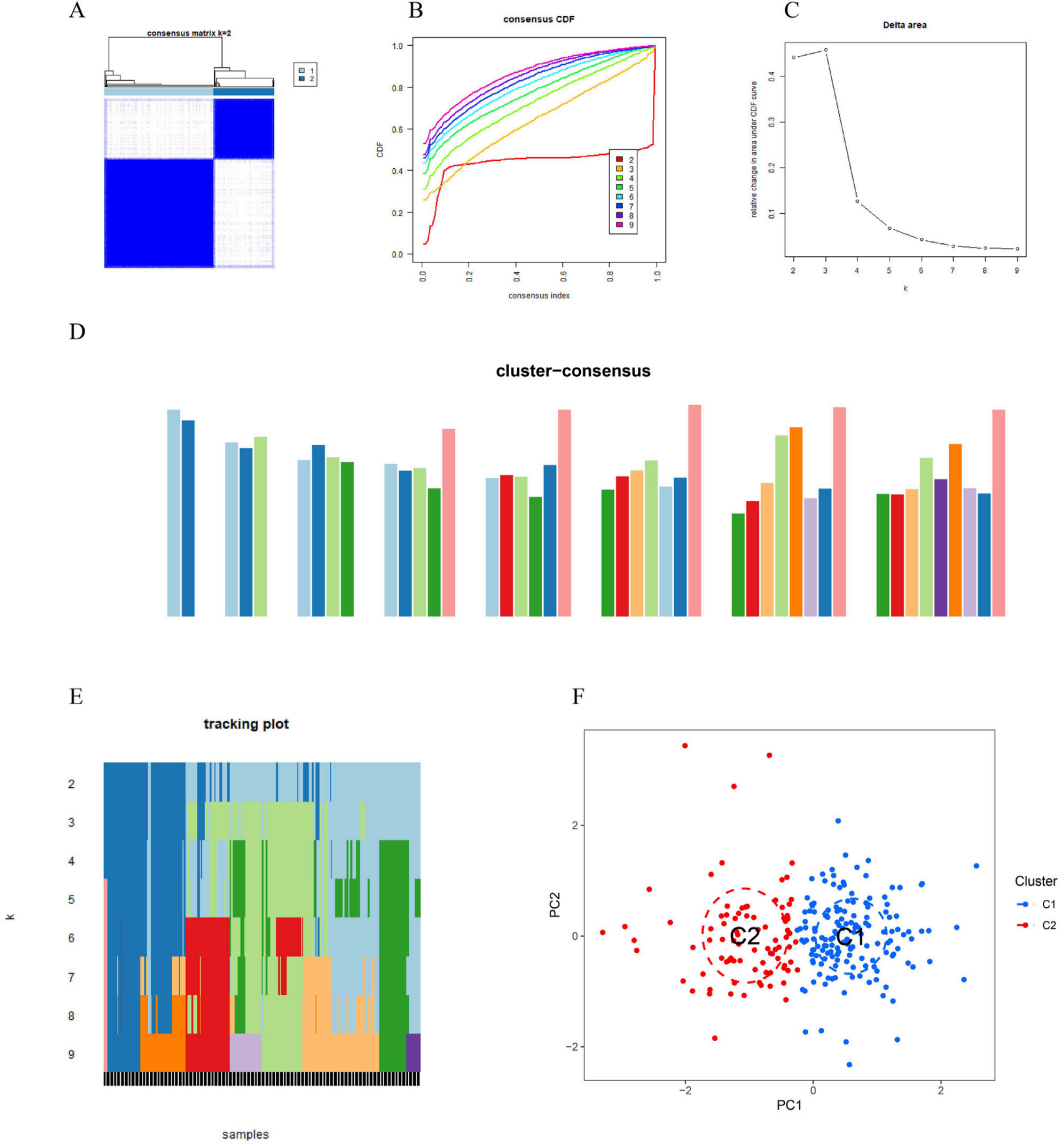
Vitamin A-related expression patterns in periodontitis (PD) were analyzed using consensus clustering. **(A, B)** Consensus clustering cumulative distribution function (CDF) curves indicate that stability was highest at k = 2, with minimal fluctuation in the consensus index range of 0.2–0.6. **(C)** Comparison of CDF curves for k values between 2 and 6 highlights differences between k and k-1. **(D)** The consistency score for each subtype exceeded 0.9 only when k = 2. **(E)** The consensus matrix heatmap shows the separation of PD patients into two distinct clusters: Cluster 1 (n = 112) and Cluster 2 (n = 198). **(F)** t-SNE analysis further confirms significant differences between the two clusters.

**FIGURE 4:**
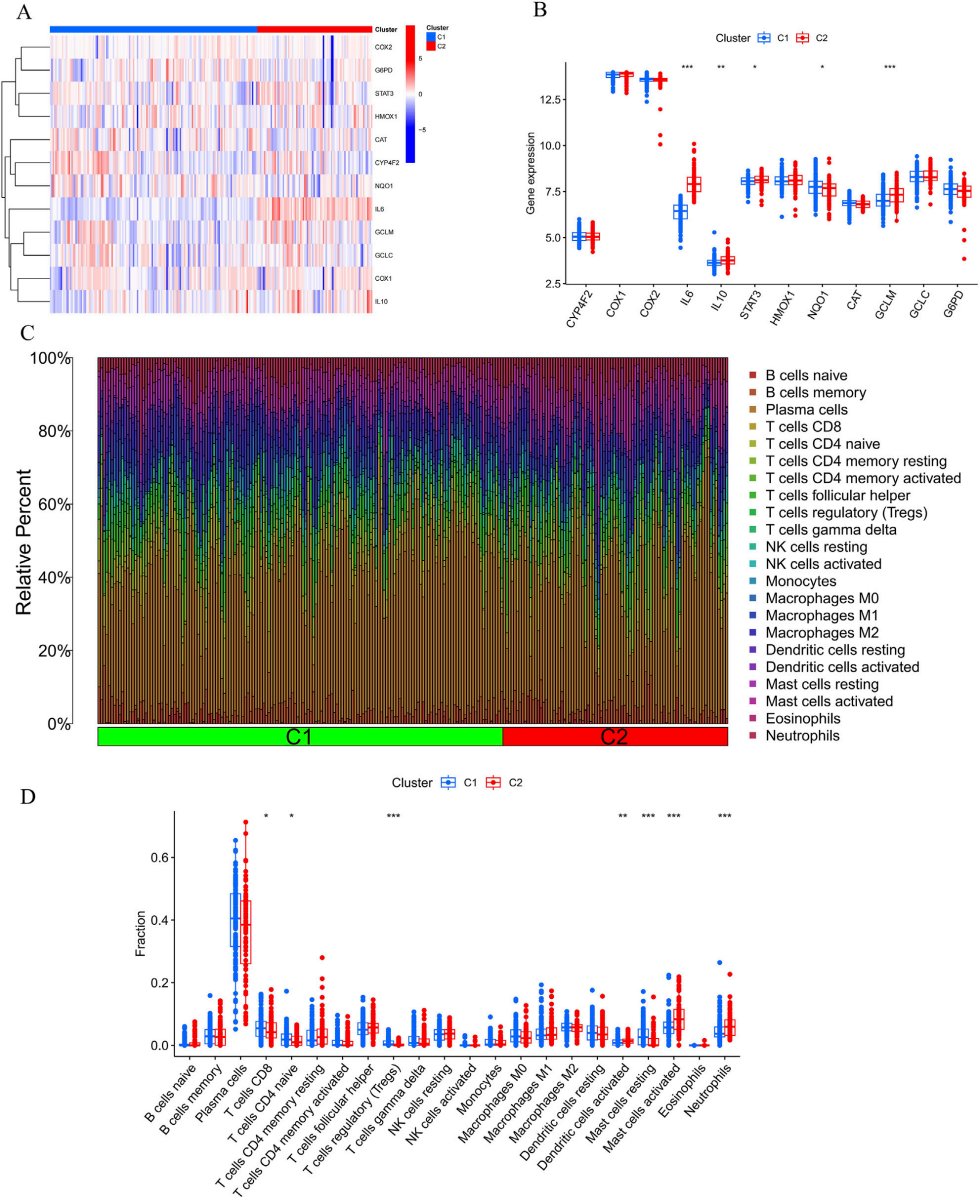
Comparative analysis of immune cell infiltration and gene expression between Cluster 1 (C1) and Cluster 2 (C2). **(A, B)** The bar plots show the relative abundance of immune cells, including B cells, T cells, NK cells, monocytes, macrophages, dendritic cells, mast cells, eosinophils, and neutrophils, with significant differences observed between C1 and C2, particularly in CD8^+^ T cells, Tregs, monocytes, and neutrophils. **(C, D)** Heatmap illustrating differential expression of genes like *COX2*, *G6PD*, *STAT3*, *IL6*, *IL10*, and others, suggesting immune pathways and Vitamin A’s potential role in modulating the response to periodontitis.

To investigate vitamin A-related expression patterns in periodontitis, PD samples were categorized using a consensus clustering algorithm based on their expression profiles. Stability was highest with a k value of 2 (k = 2), as indicated by the consensus clustering CDF curves, which showed minimal fluctuation within a consensus index range of 0.2–0.6 (Figures 3A, B). When comparing CDF curves for k values from 2 to 6, the area under the curves highlighted differences between k and k-1 (Figure 3C). Additionally, the consistency score for each subtype exceeded 0.9 only when k = 2 (Figure 3D). Therefore, PD patients were classified into two clusters: Cluster1 (n = 112) and Cluster2 (n = 198), supported by the consensus matrix heatmap (Figure 3E). t-Distributed Stochastic Neighbor Embedding (t-SNE) analysis further confirmed significant differences between these two clusters (Figure 3F). Distinct expression landscapes were observed between these two patterns (Figure 4). Interestingly, the deviation between Cluster 1 and Cluster 2 aligned with the anticipated expression patterns influenced by the presence or absence of Vitamin A stimulation, as suggested by previous research. Cluster 1, which corresponds to the anticipated expression pattern stimulated by Vitamin A, exhibited lower levels of *IL-1β*, *IL-6*, *IL-10*, *STAT3*, *HMOX1*, *GCLM* and higher levels of *NQ O -1* and *ARG-1*. This pattern suggests a protective role against the onset and progression of periodontitis. Conversely, Cluster 2, which corresponds to the expression pattern in contrast to Vitamin A stimulation, was characterized by enhanced expressions of *IL-1β*, *IL-6*, *IL-10*, *STAT3*, *HMOX1*, *GCLM* and suppressed expression of *NQ O -1* and *ARG-1* (Figures 4A, B).

Furthermore, the results of the immune infiltration analysis revealed an altered immune microenvironment between Cluster 1 and Cluster 2. This alteration was characterized by the activation of CD8^+^ T cells, regulatory T cells, monocytes, dendritic cells, mast cells, and neutrophils, which partially align with the immune response observed in the onset and progression of periodontitis based on previous data (Figures 4C, D). These findings suggest that categorizing periodontitis in terms of Vitamin A content could be beneficial, given its potential influence on the susceptibility to the onset and progression of periodontitis in an immunological context.

**FIGURE 5:**
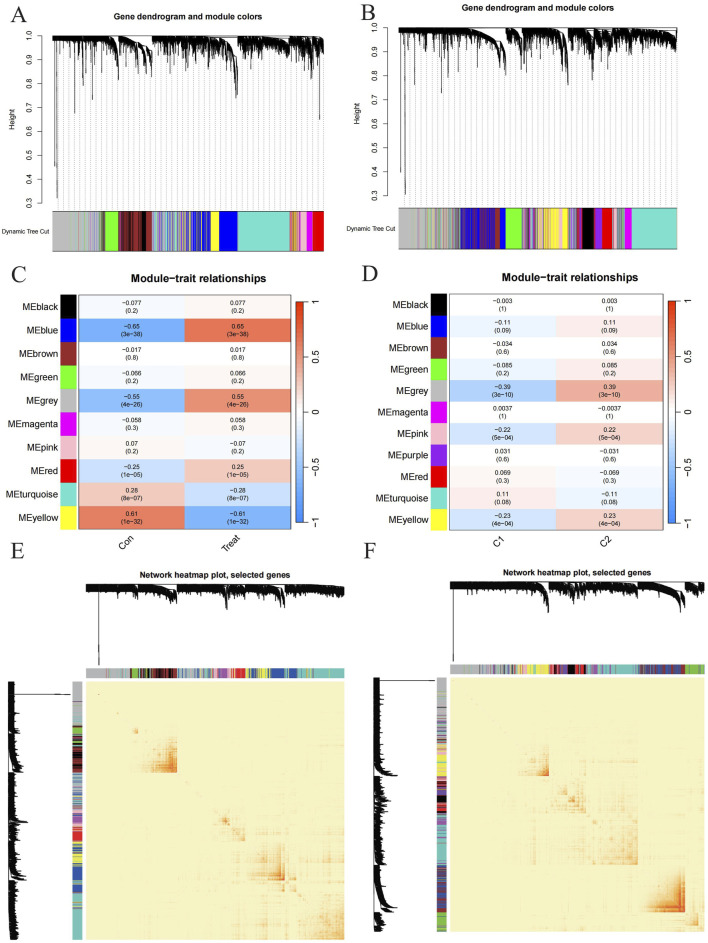
Exploration to the relationships between gene expression modules and clinical by Weighted Gene Co-expression Network Analysis (WGCNA). **(A, B)** Cluster tree dendrogram of co-expression modules among PD/control and C1/C2 respectively. **(C, D)** Correlation analysis between module eigengenes and clinical status. Each row represents a module; each column represents a clinical status among PD/control and C1/C2 respectively. **(E, F)** Representative heatmap of the correlations among modules among PD/control and C1/C2 respectively.

To identify key gene modules associated with PD, we applied the Weighted Gene Co-expression Network Analysis (WGCNA) algorithm to construct a co-expression network for normal and PD samples. Variance analysis of gene expression in GSE16134 allowed selection of the top 25% most variable genes for further analysis. Co-expressed gene modules were identified with a soft power value of 9 and a scale-free R^2 of 0.9 (Figure 5A). Using the dynamic cutting algorithm, 10 distinct co-expression modules were identified, as visualized by the heatmap of the topological overlap matrix (TOM) (Figure 5C). Analysis of module-clinical feature correlations revealed that the blue module, consisting of 999 genes, had the strongest association with PD (Figure 5E). Furthermore, a positive correlation was observed between the blue module and module-related genes.

Additionally, WGCNA was used to analyze critical gene modules related to PD clusters. The optimal soft threshold parameters were β = 7 and R^2 = 0.9, which facilitated the construction of a scale-free network (Figure 5B). Eleven significant modules were identified, and their TOMs were depicted in the heatmap (Figure 5C). Correlation analysis between modules and PD revealed a strong association between the grey module (containing 1,661 genes) and PD clusters (Figure 5E). This analysis also indicated a significant relationship between turquoise module genes and the selected module.

### 3.3 Identification of cluster-specific DEGs and functional annotation

**FIGURE 6:**
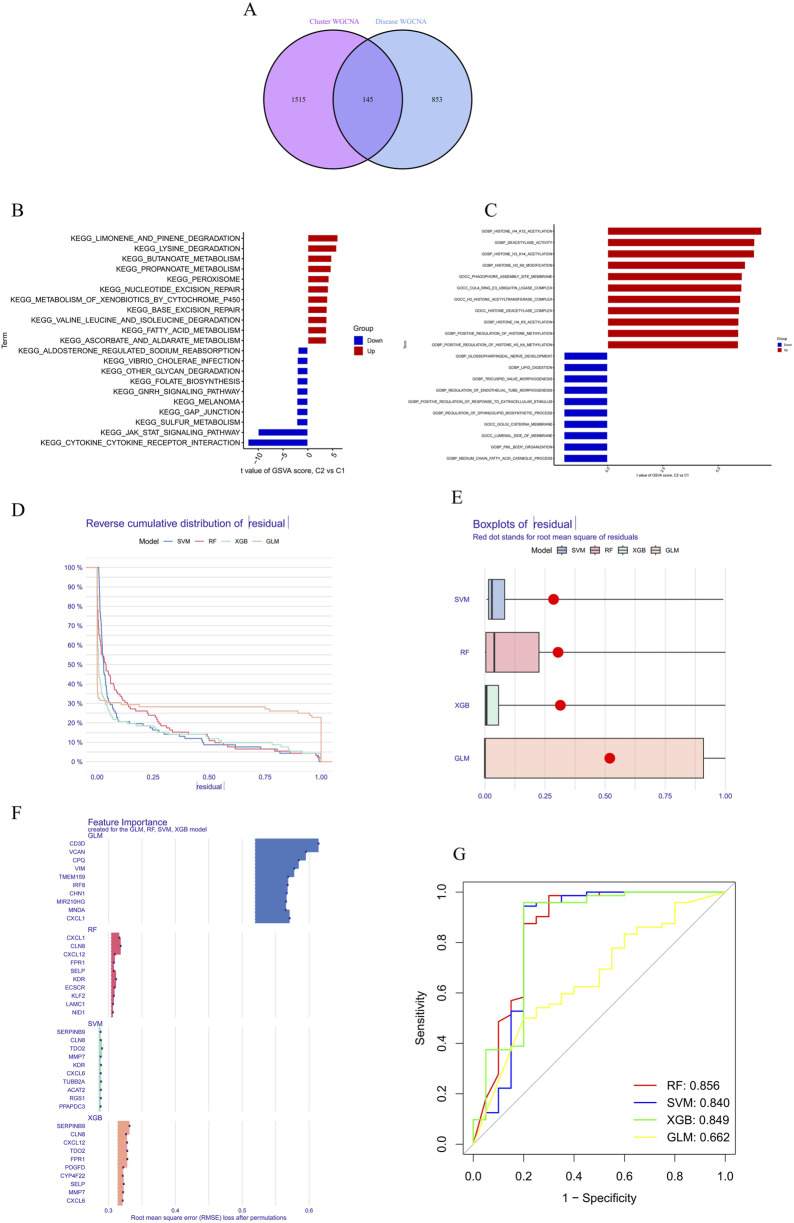
Differential expression and pathway analysis between C2 and C1 groups and machine learning. **(A)** Functional differences between clusters were analyzed using GSVA, revealing that the KEGG_JAK_STAT_SIGNALING_PATHWAY was upregulated in the C2 group. **(B)** Metabolic pathway analysis demonstrated significant differences in the C2 group in terms of fatty acid metabolism, indicating potentially higher metabolic capacity in the C2 group. **(C)** Functional enrichment analysis revealed differences in biological processes and cellular components between the C1 and C2 groups. **(D, E)** Residual distribution comparison of four machine learning models (RF, SVM, GLM, XGB) showed that the SVM and XGB models had lower residuals, indicating better fit. **(F)** RMSE was used to assess the importance of model feature variables, identifying the top 15 important variables in the XGB model. **(G)** The ROC curve showed that the RF model performed best in the test set, with an AUC of 0.856.

A total of 145 cluster-specific differentially expressed genes (DEGs) were identified by analyzing the intersections between module-related genes of ATGs-related clusters and those related to PD and non-PD individuals (Figure 6A). Gene Set Variation Analysis (GSVA) was used to explore functional differences between the clusters. The analysis revealed that the KEGG_JAK_STAT_SIGNALING_PATHWAY has a positive t-value, indicating its upregulation in the C2 group. The JAK-STAT signaling pathway plays a critical role in cell proliferation, differentiation, apoptosis, and immune regulation, suggesting that C2 may have enhanced activity in these processes, indicative of metabolic reprogramming in signal transduction. Additionally, other metabolic pathways, such as fatty acid metabolism (KEGG_FATTY_ACID_METABOLISM) and amino acid degradation (e.g., KEGG_VALINE_LEUCINE_AND_ISOLEUCINE_DEGRADATION), showed distinct enrichment patterns, suggesting significant metabolic differences between C1 and C2. These differences imply that the C2 group may have a higher capacity for fatty acid and amino acid metabolism, potentially linked to altered physiological or pathological states (Figure 6B).

Functional enrichment analysis further highlighted differences in biological processes and cellular components between C1 and C2, particularly in areas of metabolic reprogramming and epigenetic regulation (Figure 6C). The C2 group exhibited upregulation in lipid metabolism-related processes, such as “GOBP_MEDIUM_CHAIN_FATTY_ACID_CATABOLIC_PROCESS” and “GOBP_LIPID_DIGESTION,” indicating a greater reliance on fatty acid metabolism and lipid digestion for energy production or cellular restructuring. Moreover, C2 showed significant upregulation in epigenetic modification processes, including “GOBP_POSITIVE_REGULATION_OF_HISTONE_H3_K4_METHYLATION,” “GOBP_HISTONE_H4_K5_ACETYLATION,” and “GOBP_HISTONE_H3_K14_ACETYLATION,” suggesting that C2 might use these modifications to regulate gene expression in response to different cellular states or environmental conditions. The C2 group also exhibited distinct expression patterns related to cellular components such as “GOCC_GOLGI_CISTERNA_MEMBRANE” and “GOCC_PHAGOPHORE_ASSEMBLY_SITE_MEMBRANE,” which could be linked to alterations in secretory activities, membrane transport, or autophagy. Enhanced regulation of developmental processes, including “GOBP_REGULATION_OF_ENDOTHELIAL_TUBE_MORPHOGENESIS” and “GOBP_TRICUSPID_VALVE_MORPHOGENESIS,” in the C2 group further suggests metabolic reprogramming linked to specific tissue or organ development.

**FIGURE 7:**
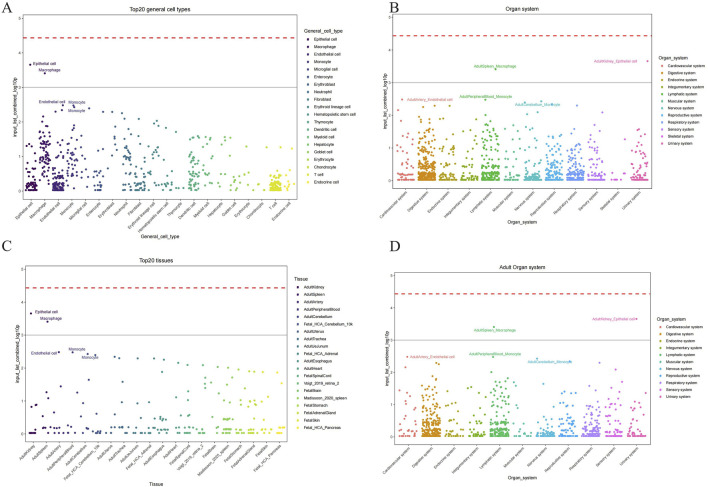
Cell types identified by CSEA analysis across various tissues. **(A)** The major cell types encompassed epithelial cells, endothelial cells, macrophages, monocytes, and several others across different tissues. **(B)** Maped top 20 general cell types and their relative abundance. **(C)** Organ system-specific analysis delineating the presence of these cell types across mapped on the cellular composition and potential functional dynamics in these systems. **(D)** Maped top 20 general cell types and their relative abundance in adult organ system.

We conducted the CSEA (Cell-specific Enrichment Analysis) by processing the gene expression data from periodontitis samples to identify pathways specifically enriched in macrophages. The analysis focused on determining the association between the JAK-STAT pathway and mitochondrial metabolic reprogramming within these immune cells. This approach allowed us to rigorously pinpoint the pathways of interest, demonstrating that the JAK-STAT pathway plays a critical role in the metabolic alterations observed in macrophages during periodontitis (Figure 7).

Overall, the primary differences between C1 and C2 are associated with lipid metabolism, epigenetic modifications, cellular organelle structure, and development-related processes. The unique metabolic reprogramming of the C2 group, characterized by upregulation of lipid metabolism and epigenetic regulation pathways, reflects its adaptation to meet distinct cellular functional requirements.

### 3.4 Construction and assessment of machine learning models

To identify subtype-specific genes with high diagnostic value, four established machine learning models—Random Forest (RF), Support Vector Machine (SVM), Generalized Linear Model (GLM), and eXtreme Gradient Boosting (XGB)—were developed based on the expression profiles of 909 cluster-specific DEGs in the PD training cohort (PD samples randomly divided into a training cohort (70%) and a validation cohort (30%) as mentioned). The “DALEX” package was utilized to interpret these models and visualize the residual distribution for each model in the test set. The SVM and XGB models exhibited relatively lower residuals (Figures 6D, E). The top 15 important feature variables of each model were ranked according to root mean square error (RMSE) (Figure 6F).

The discriminative performance of the four models was further evaluated in the testing set using receiver operating characteristic (ROC) curves based on 5-fold cross-validation. The RF model demonstrated the highest area under the ROC curve (AUC) (GLM, AUC = 0.662; SVM, AUC = 0.840; RF, AUC = 0.856; XGB, AUC = 0.849) (Figure 6G). Overall, the XGB model was shown to be the most effective in distinguishing patients with different clusters. The top five most important variables identified by the XGB model (RGS1, ACAT2, KDR, TUBB2A, and TDO2) were selected for further analysis.

**FIGURE 8:**
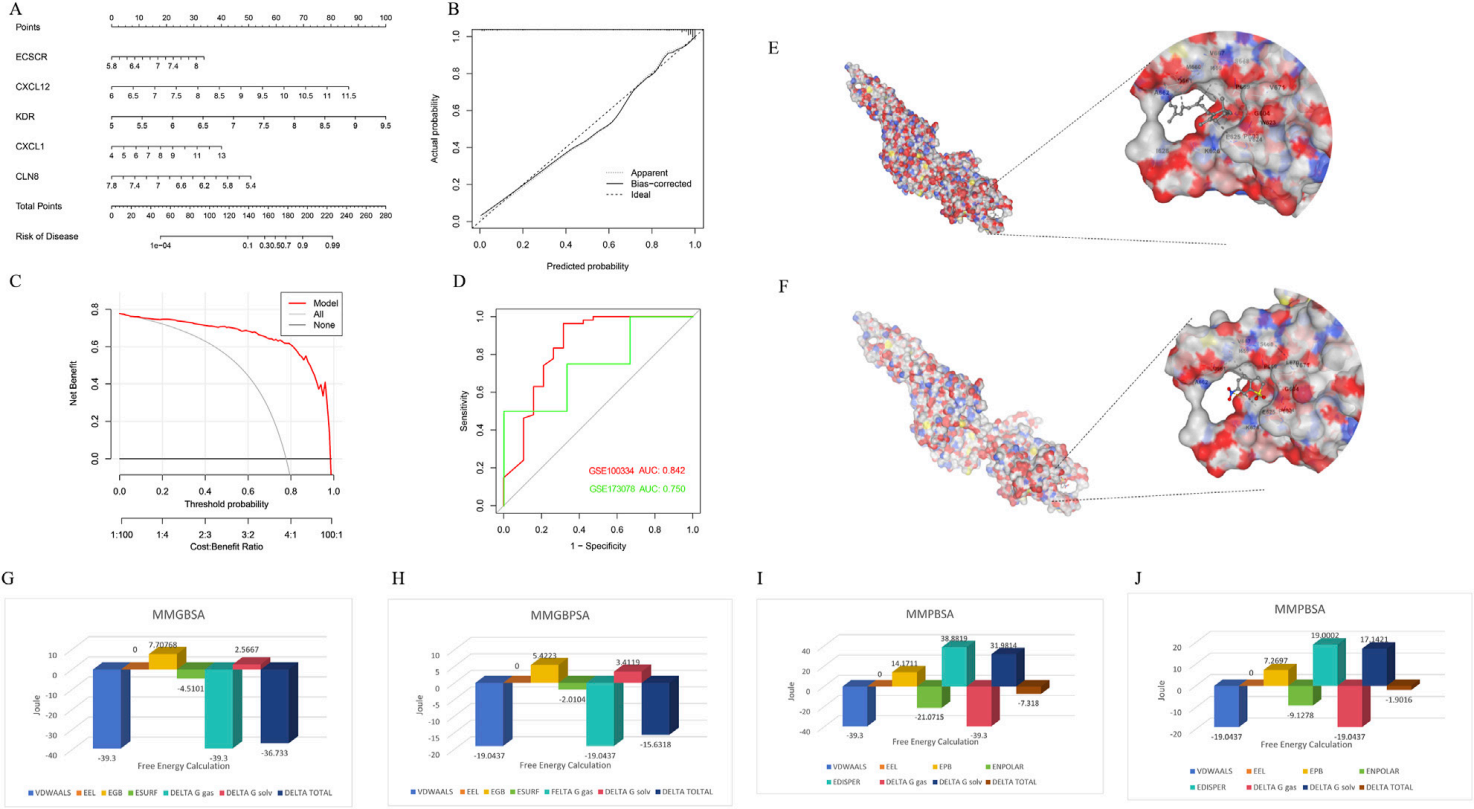
Confirmation to diagnostic value of XGB model and molecular simulation. **(A)** A nomogram was constructed to evaluate the predictive efficiency of the XGB model for estimating the risk of PD clusters in patients. **(B)** The calibration curve demonstrated minimal error between actual and predicted PD cluster risks, indicating high prediction accuracy. **(C)** The decision curve analysis (DCA) suggested that the nomogram has high accuracy and may serve as a useful tool for clinical decision-making. **(D)** External datasets have confirmed the consistent diagnostic value of these markers. **(E)** CB-DOCK2 molecular docking and AMBER22 molecular dynamics simulations revealed that vitamin A can bind near the phosphorylation site of STAT3 (PDB ID: 6NJS), mimicking the effect of the inhibitor Stattic and interfering with STAT3 phosphorylation. **(F)** Stattic had a vina score of −4.9, showing similar binding sites but slightly weaker binding affinity. **(G–J)** Further MMGBSA and MMPBSA analyses indicated that vitamin A had a more negative total free energy (ΔG), reflecting stronger binding stability and higher inhibitory potential on STAT3 compared to Stattic.

To assess the predictive efficiency of the XGB model, a nomogram was constructed to estimate the risk of PD clusters in patients (Figure 8A). The predictive accuracy of the nomogram was evaluated using a calibration curve and decision curve analysis (DCA). The calibration curve indicated minimal error between the actual and predicted risk of periodontitis clusters (Figure 8B), while the DCA suggested that the nomogram has high accuracy, potentially serving as a useful tool for clinical decision-making (Figure 8C). ROC-curve based on external dataset GSE10334 (Figure 8D) and GSE173078 (Figure 8D) illustrated these markers has acceptable accuracy and may serve as a useful tool for clinical decision-making.

### 3.5 Vitamin A reshapes mitochondrial metabolic reprogramming in inflammation-associated macrophages through the JAK-STAT pathways

Through CB-DOCK2 molecular docking and AMBER22 molecular dynamics simulations, it was discovered that vitamin A can bind near the phosphorylation site of STAT3, mimicking the effect of the inhibitor Stattic and interfering with STAT3 phosphorylation. Vitamin A achieved a vina score of −5.6, indicating a relatively stable binding, with key residues including PRO603, GLY604 (PRO603 GLY604 TRP623 VAL624 GLU625 LYS626 ASP627 ILE628 GLN633 GLN635 SER636 VAL637 GLU638 TYR657 ILE659 MET660 ASP661 ALA662 THR663 VAL667 SER668 PRO669 LEU670 VAL671), and TRP623 (Figure 8E). In comparison, Stattic had a vina score of −4.9, with similar binding sites but slightly weaker binding affinity (PRO603 GLY604 TRP623 VAL624 GLU625 LYS626 ASP627 ILE628 ILE659 MET660 ASP661 ALA662 THR663 VAL667 SER668 PRO669 LEU670 VAL671) (Figure 8F). Further MMGBSA and MMPBSA analyses revealed that vitamin A had a more negative total free energy (ΔG), showing stronger binding stability and favorable binding energy (ΔG of −36.733 kcal/mol), while Stattic had a ΔG of −15.6318 kcal/mol, indicating relatively weaker interactions (Figures 8G–J). Detailed MMPBSA/MMGBSA results and standard deviations can found in supplementary material (Supplementary Tables S2–S5). These findings suggest that vitamin A may have stronger inhibitory potential on STAT3 at the molecular level and could interfere with its function through stable binding near the phosphorylation site.

**FIGURE 9:**
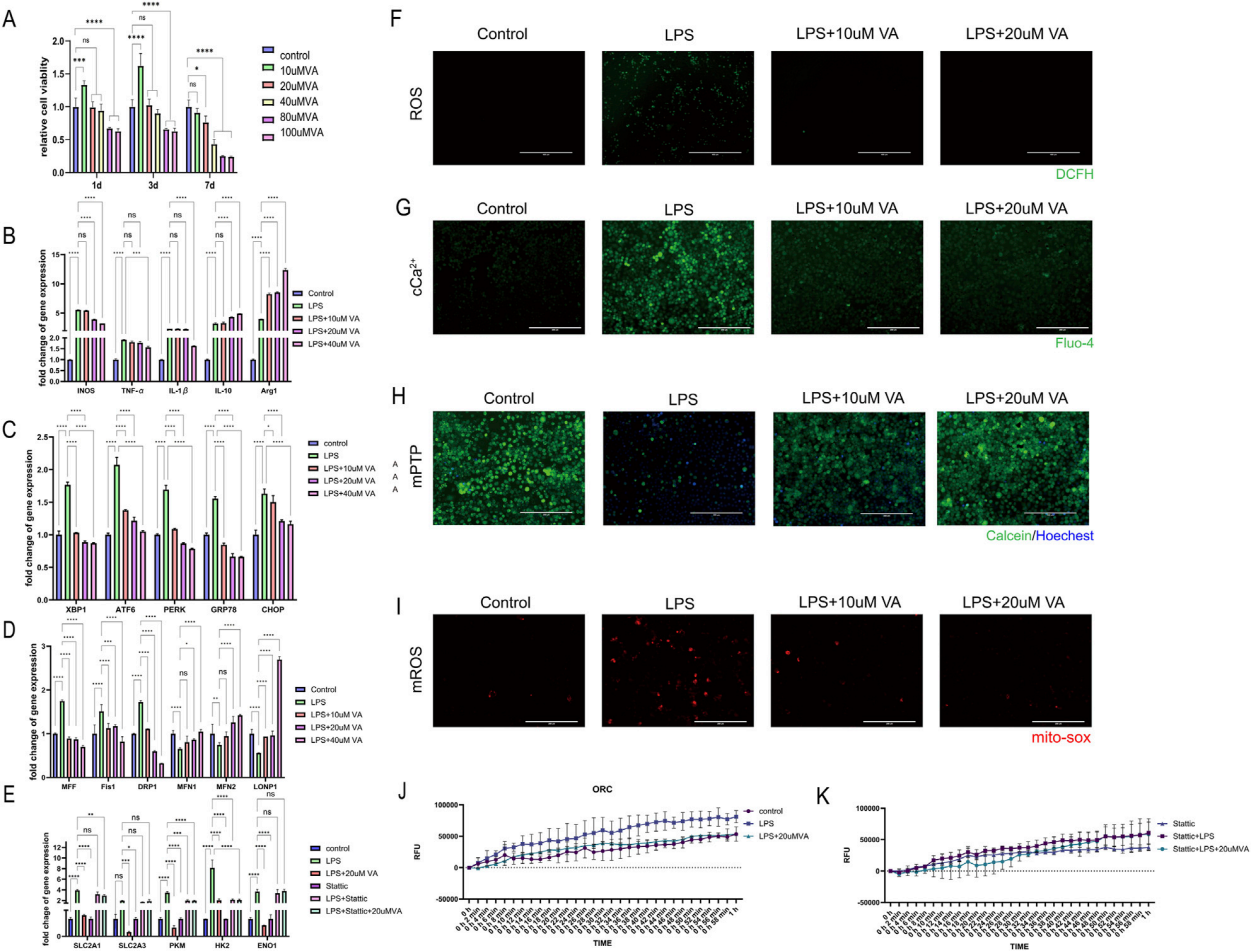
*In vitro* validation of the effect of VA on macrophages. **(A)** CCK-8 cell viability assay was used to observe the cytotoxicity of VA dissolved in DMSO at different concentrations. **(B–D)** Expression of genes related to inflammation, ER stress, and mitochondrial dynamics in RAW264.7 treated with PBS, LPS, and LPS + VA. **(F)** DCFH fluorescence staining was used to detect the ROS content in RAW264.7 under different treatments. Scale bar = 400 µm. **(E)** Gene expression levels related to glycolysis were evaluated in RAW264.7 cells in state of inflammation and VA supplement treated with Stattic **(G)** Flou-4 fluorescence staining was used to detect the Ca2+ content in RAW264.7 under different treatments. Scale bar = 200 µm. **(H)** Calcein-AM loading/CoCl2 quenching assay was used to detect the opening of mPTP in RAW264.7 under different treatments. Scale bar = 200 µm. **(I)** MitoSOX fluorescence staining was used to detect mitochondrial superoxide levels in RAW264.7 cells under various treatments. Scale bar = 200 µm. All quantitative results are expressed as mean ± SD (n = 3). **(J, K)** Oxygen consumption rate was measured to estimate the mitochondrial functional state of metabolism.

The impact of different concentrations of VA dissolved in DMSO on the survival rate of macrophages (RAW264.7) was accessed using the CCK-8 cell viability assay, preliminarily validating the safety and toxicity of VA (Figure 9A). This step laid the foundation for subsequent research by ensuring that VA does not exhibit significant toxicity at appropriate concentrations.

Further investigation to the regulatory role of VA on the inflammatory response of macrophages in the context of periodontitis. As ER stress and mitochondrial dynamics are key factors influencing macrophage metabolic reprogramming in response to immune challenges, by analyzing gene expression related to inflammatory responses, ER stress, and mitochondrial dynamics (Figures 9B–D), We found that LPS significantly induced inflammatory responses and induced the polarization of macrophages toward the M1 type, while causing significant endoplasmic reticulum stress and abnormal mitochondrial dynamics. VA significantly regulated the expression of these genes and inhibited the inflammatory response of macrophages. Promote their differentiation to M2 type, improve the endoplasmic reticulum stress state of cells, inhibit mitochondrial fission, promote mitochondrial fusion and the stability of mitochondria-endoplasmic reticulum complex, thereby changing the metabolic reprogramming of macrophages. Six experimental groups were established to estimate the presence of STAT3 is a key factor for vitamin A to exert its protective effects (Figure 9E): the Control group, the lipopolysaccharide (LPS) group, the LPS combined with vitamin A group (LPS + VA), the STAT3 inhibitor group (Stattic), the LPS combined with Stattic group (LPS + Stattic), and the LPS, Stattic, and 20 μM vitamin A combined treatment group (LPS + Stattic + 20 μMVA). Through PCR analysis of glycolysis-related genes (*SLC2A1*, *SLC2A3*, *PKM*, *HK2*, and *ENO1*) and oxidative phosphorylation (OCR) experiments to assess mitochondrial function, we verified the effect of vitamin A on mitochondrial metabolic reprogramming through STAT3 regulation under periodontitis conditions. PCR analysis showed that in the LPS-treated group, the expression of glycolysis-related genes *SLC2A1*, *SLC2A3*, *PKM*, *HK2*, and *ENO1* was significantly upregulated, indicating that LPS induced the activation of the glycolytic pathway and metabolic reprogramming. In the LPS + VA group, the expression levels of these genes significantly decreased, approaching those of the control group, suggesting that vitamin A effectively inhibits LPS-induced metabolic dysregulation, indicating its protective role in maintaining metabolic homeostasis under inflammatory conditions.

Additionally, oxidative stress is considered a major driving force behind mitochondrial dysfunction in the periodontitis process. To evaluate the potential role of VA in this process, the researchers detected intracellular reactive oxygen species (ROS) levels using the DCFH fluorescence staining method (Figure 9F). The results showed that under LPS-induced periodontitis conditions, ROS levels were significantly elevated, while VA treatment effectively reduced ROS content, indicating that VA alleviates oxidative stress. The mitigation of oxidative stress is closely related to mitochondrial metabolic reprogramming, suggesting that VA may influence mitochondrial function by regulating ROS production.

Calcium homeostasis is another important regulator of mitochondrial function and cellular metabolism. Changes in intracellular calcium levels was examined using the Fluo-4 fluorescence staining method (Figure 9G), and the results indicated that VA plays a regulatory role in maintaining calcium levels during LPS-induced calcium homeostasis dysregulation. This finding further supports the potential role of VA in metabolic reprogramming, as calcium homeostasis is critical for mitochondrial health and metabolic function. To further assess the protective effects of VA on mitochondrial dysfunction, we detected the opening of the mitochondrial permeability transition pore (mPTP) using the Calcein-AM/CoCl2 quenching assay (Figure 9H). The results showed that LPS treatment induced excessive mPTP opening in periodontitis macrophages, while VA treatment significantly inhibited this process, suggesting that VA helps maintain mitochondrial membrane integrity and prevents mitochondrial dysfunction.

Regulation of reactive oxygen species (ROS) levels is another crucial aspect of mitochondrial function and cellular metabolism. To further assess the protective effects of VA on mitochondrial dysfunction, we used MitoSOX to measure mitochondrial superoxide generation (Figure 9I). The results showed that LPS treatment led to a significant increase in superoxide levels in periodontitis macrophages, while VA treatment markedly inhibited this process, suggesting that VA helps maintain mitochondrial redox balance and prevents mitochondrial dysfunction. This finding further supports the potential role of VA in metabolic reprogramming, as maintaining the balance of superoxide levels is critical for mitochondrial health and metabolic function.

Oxygen consumption rate (OCR) experimental results showed that LPS treatment significantly reduced the oxygen consumption rate of mitochondria, indicating that LPS impaired mitochondrial function, leading to a decline in cellular energy metabolism. However, in the LPS + VA group, OCR significantly recovered, suggesting that vitamin A can partially reverse LPS-induced mitochondrial damage, restoring mitochondrial oxidative phosphorylation function to support normal cellular metabolic demands (Figure 9J).To elucidate the role of the STAT3 signaling pathway in this metabolic reprogramming, we introduced Stattic, a specific STAT3 inhibitor. Stattic alone did not induce significant metabolic changes, possibly because STAT3 was not fully activated in the non-inflammatory environment. However, in the LPS + Stattic group, the expression of metabolic genes and mitochondrial function were further reduced compared to the LPS group, indicating that STAT3 plays a protective role under inflammatory conditions by regulating the expression of metabolic genes to maintain mitochondrial function (Figure 9K). Crucially, in the LPS + Stattic + 20 μMVA group, the expression levels of metabolic genes and OCR were not significantly different from those in the LPS + Stattic group, indicating that when the STAT3 signaling pathway is inhibited, vitamin A cannot further inhibit the expression of metabolic genes and mitochondrial function. This result confirms that vitamin A regulates metabolic reprogramming through the STAT3 pathway, and the presence of STAT3 is a critical link for the protective effects of vitamin A.

## 4 Discussion

Vitamin A is well-known for its crucial role in maintaining epithelial integrity and modulating immune responses (Polcz and Barbul, 2019). However, its specific effects on chronic periodontitis, particularly through its influence on metabolic pathways, have not been thoroughly explored. This study is among the first to elucidate how vitamin A might impact inflammatory processes and metabolic reprogramming in periodontal tissues (Golebski et al., 2021). By demonstrating how vitamin A influences the JAK-STAT pathway in macrophages, leading to mitochondrial metabolic reprogramming, the research provides new insights into leveraging vitamin A for more effective management of periodontitis. The findings suggest that vitamin A could help modulate immune responses, reduce inflammation, and potentially slow the progression of periodontal disease.

These findings indicate that incorporating vitamin A or its derivatives into treatment protocols could enhance the effectiveness of existing periodontal therapies by addressing the underlying metabolic dysregulation that contributes to chronic inflammation. This approach could lead to more personalized treatment strategies that consider a patient’s nutritional status and specific metabolic needs.

This study, using bioinformatics analysis validation, identified a strong correlation between 1,661 genes in the gray module and periodontitis clusters in inflamed tissue samples from periodontitis patients. Additionally, the study employed WGCNA (Weighted Gene Co-expression Network Analysis) to construct a scale-free network, analyzing the correlation between gene modules and clinical traits, further confirming the importance of these genes in periodontitis. Notably, the JAK-STAT pathway plays a central role in regulating macrophage metabolism, and the regulatory effect of vitamin A is likely mediated through this pathway. CSEA (Cell-specific Enrichment Analysis) further revealed the metabolic characteristics of macrophages in periodontitis, particularly the differences between C1 and C2 clusters. The upregulation of lipid metabolism and epigenetic regulation in the C2 cluster indicates specific metabolic reprogramming in macrophages within a particular environment, further highlighting the critical role of vitamin A in these metabolic changes.

Through *in vitro* experiments, the study demonstrated that the vitamin A family regulates mitochondrial metabolic reprogramming in macrophages via the JAK-STAT signaling pathway, thereby inhibiting the progression of periodontitis. Specifically, the antioxidant effects of vitamin A alter the metabolic state of macrophages, reduce mitochondrial stress, and change mitochondrial dynamics through the JAK-STAT pathway, ultimately suppressing inflammation, deepening our understanding of inflammation and metabolic reprogramming, and offering new biomarkers and therapeutic targets for future personalized treatment strategies.

Beyond the therapeutic implications, this study also deepens the understanding of the genetic and immunological factors involved in periodontitis. The research identified two phenotypes related to genetic susceptibility and immune infiltration and discovered five key genetic markers (*RGS1*, *ACAT2*, *KDR*, *TUBB2A*, and *TDO2*), highlighting the complex interplay between genetics, immune response, and metabolic reprogramming in the development of periodontitis. These findings suggest that vitamin A might influence the expression and function of these genetic markers, thereby affecting an individual’s susceptibility to periodontitis. Among these markers, the activity of *ACAT2* is closely related to mitochondrial metabolism because it is involved in fatty acid metabolism. Changes in fatty acid metabolism can affect mitochondrial function, thereby influencing the metabolic reprogramming and inflammatory response of immune cells (Wang et al., 2017). *TUBB2A* is involved in the assembly of the cell cytoskeleton, and changes in its expression may affect the positioning and function of mitochondria within the cell, thus impacting mitochondrial metabolic reprogramming (Sferra et al., 2018). The activity of *TDO2* is also closely related to mitochondrial metabolism, as alterations in tryptophan metabolism can influence mitochondrial energy metabolism and oxidative stress. These metabolic changes may affect the activity and inflammatory response of immune cells by modulating mitochondrial function (Lee et al., 2022). Future research could further explore how these genetic factors interact with vitamin A metabolism, as well as mitochondrial metabolic reprogramming, potentially leading to more targeted interventions based on a patient’s genetic profile.

While this study provides important insights into the potential role of vitamin A in periodontitis, further research is needed to validate these findings and explore their broader implications. Like other applicable periodontitis datasets, GSE173078 has issues with inconsistent diagnostic criteria compared to the training dataset (GSE16134) and an insufficient sample size for periodontal disease. These inconsistencies and limitations could potentially lead to misleading interpretations of the diagnostic value of the selected marker genes. Despite these challenges, GSE16134, which has consistent diagnostic criteria and an adequate sample size, serves as a more reliable external validation dataset. While GSE16134 remains representative, it is also important to note that it originates from the same research center as the training dataset, which may introduce certain level of dependency. Multi-center studies are needed to better understand the findings and enhance their generalizability. Future studies could investigate the effects of vitamin A on other types of immune cells and in different inflammatory environments to better understand its full therapeutic potential (Varela-López et al., 2018). Additionally, clinical trials are necessary to assess the safety and efficacy of vitamin A-based interventions in diverse patient populations (Ross, 2002). Regarding the further promotion of vitamin A supplementation, different vitamin A derivatives (such as retinol and retinoic acid) may exhibit different effects in various inflammatory environments, requiring further research. Additionally, individual differences among patients (such as genetic background, lifestyle, and dietary habits) can influence the absorption and metabolism of vitamin A. Therefore, clinical trials need to consider these factors to ensure the reliability and generalizability of the results. The ultimate goal is to integrate nutritional interventions with traditional periodontal therapies to develop a more comprehensive and effective approach to managing periodontitis.

## 5 Conclusion

In summary, this study significantly enhances our understanding of how the vitamin A family might influence the pathogenesis of periodontitis through its effects on mitochondrial metabolic reprogramming in macrophages via the JAK-STAT pathway. These findings provide a strong foundation for future research and clinical applications, suggesting that vitamin A could be a valuable addition to the arsenal of treatments available for managing periodontitis. By offering new insights into the mechanisms underlying periodontitis, this research opens promising avenues for improving patient outcomes through more targeted and personalized therapeutic strategies.

## Data Availability

The data presented in the study are deposited in the Gene Expression Omnibus repository, accession numbers GSE16134, GSE173078, GSE10334.
