# Supplementary material for: Vitamin A family suppresses periodontitis by restoring mitochondrial metabolic reprogramming in macrophages through JAK-STAT pathway
Source: Front Genet. 2025 Jan 28;16:1505933. doi: 10.3389/fgene.2025.1505933 (PMC11810908; doi:10.3389/fgene.2025.1505933)
Supplement: Supplementary file 1 [file Table1.docx]

| **Gene** | **Forward Primer Sequence (5ʹ-3ʹ)** | **Reverse Primer Sequence (5ʹ-3ʹ)** |
| --- | --- | --- |
| GAPGH | CAAGAAGGTGGTGAAGC | GGAAGAGTGGGAGTT |
| GRP75 | ACATCAACTTGCCATACCTTACC | ATGCCACCAACCAGAATCAC |
| IP3R | CAGAGGTAGACCCTGACTTTGAG | GATGGAGGAGATGTCGTTGC |
| MCU  Vdac1  ATF6  PERK  Xbp1  CHOP  GPR78  Fis1  Mff  DRP1  Mfn1  Mfn2  Lonp1  Cox1  IL1b  IL6  IL10  STAT3  NQO1  CAT  GCLM  ARG1 | CACCAGATGGCGTTCGAGTC  TGACGGGACAGAGTTTGGTG  CTTTCCGTGACTAAACCTGTTCTAC  GCACTGGATGCCGAGAATG  CAAGTGGTGGATTTGGAAGAAG  AGGTCCTGTCCTCAGATGAAATTG  AGCCAACTGTAACAATCAAGGTC  AACAGCGGGACTATGTCTTCTAC  CACCACCAAATGCTGACCTG  TGCCCGAGAACAGCGAGAT  CAACTCCTACTGCTCCTTCTAACC  ATGGGCATTCTTGTGGTCG  AGCCCTATGTTGGCGTCTTC  CTGTGAGGCACCAGTCTGAA  AAGGGGCAAGAAGAAGTAGCAG  ACCTTCCAAAGATGGCTGAA  TTTCCCTGACCTCCCTCTAAT  TTCTCCTTCTGGGTCTGGCT  GTGGAGTCGGACCTCTATGC  CCTATCCTGACACTCACCGC  TCCTTGGAGCATTTACAGCCT  TCCCTGGGGAAGACACCAGAA | GGGTCTTCACGTCGTTCAGC  CTGCTATTCCGAAGCGAGTG  CAGACTCTCGGTTCTTTATCATCC  GCTTGCTGAGGCTAGATGAAAC  TCCATTCCCAAGCGTGTTC  GGCTTTGGGATGTGCGTGT  GCTGTCACTCGGAGAATACCAT  CCTTATCAATCAGGCGTTCC  CCTGTAACCACGATCCTCTCTG  GCATTACTGCCTTTGGGACACT  AGGGACGCCAATCCTGTGA  GCTTCTCACTGGCGTATTCC  GCGAATGTTCCCGTATGGTAG  TATGCGCCCACAAACTGGAA  AGAGAGCACACCAGTCCAAA  TGGCTTGTTCCTCACTACTCT  GCTCCCTGGTTTCTCTTCC  GCCTCCTTCTTTGCTGCTTTC  GGGTCCTTCAGTTTACCTGTGA  GAGCACGGTAGGGACAGTTC  ACACAGCAGGAGGCAAGATT  TCCCGAGCAAGTCCGAAACAA |

**Table 1 Synthetic primer sequences**

**Table 2 MMGBSA result of VA (ligand) and STAT3**

| Energy Component | Average | Std. Dev. | Std. Err. of Mean |
| --- | --- | --- | --- |
| VDWAALS | -39.3 | 2.8338 | 0.2892 |
| EEL | 0 | 0 | 0 |
| EGB | 7.0768 | 1.3585 | 0.1386 |
| ESURF | -4.5101 | 0.2151 | 0.022 |
| DELTA G gas | -39.3 | 2.8338 | 0.2892 |
| DELTA G solv | 2.5667 | 1.3434 | 0.1371 |
| DELTA TOTAL | -36.7333 | 2.5377 | 0.259 |

**Table 3 MMPBSA result of VA (ligand) and STAT3**

| Energy Component | Average | Std. Dev. | Std. Err. of Mean |
| --- | --- | --- | --- |
| VDWAALS | -39.3 | 2.8338 | 0.2892 |
| EEL | 0 | 0 | 0 |
| EPB | 14.1711 | 1.9807 | 0.2022 |
| ENPOLAR | -21.0715 | 1.1475 | 0.1171 |
| EDISPER | 38.8819 | 1.3731 | 0.1401 |
| DELTA G gas | -39.3 | 2.8338 | 0.2892 |
| DELTA G solv | 31.9814 | 2.4229 | 0.2473 |
| DELTA TOTAL | -7.3186 | 2.9761 | 0.3038 |

**Table 4 MMGBSA result of Stattic (ligand) and STAT3**

| Energy Component | Average | Std. Dev. | Std. Err. of Mean |
| --- | --- | --- | --- |
| VDWAALS | -19.0437 | 2.0586 | 0.2101 |
| EEL | 0 | 0 | 0 |
| EGB | 5.4223 | 0.9485 | 0.0968 |
| ESURF | -2.0104 | 0.1669 | 0.017 |
| DELTA G gas | -19.0437 | 2.0586 | 0.2101 |
| DELTA G solv | 3.4119 | 0.8912 | 0.091 |
| DELTA TOTAL | -15.6318 | 1.8693 | 0.1908 |

**Table 5 MMPBSA result of Stattic (ligand) and STAT3**

| Energy Component | Average | Std. Dev. | Std. Err. of Mean |
| --- | --- | --- | --- |
| VDWAALS | -19.0437 | 2.0586 | 0.2101 |
| EEL | 0 | 0 | 0 |
| EPB | 7.2697 | 2.3062 | 0.2354 |
| ENPOLAR | -9.1278 | 0.8523 | 0.087 |
| EDISPER | 19.0002 | 1.0435 | 0.1065 |
| DELTA G gas | -19.0437 | 2.0586 | 0.2101 |
| DELTA G solv | 17.1421 | 2.3458 | 0.2394 |
| DELTA TOTAL | -1.9016 | 2.5902 | 0.2644 |

**Table 6 Important genes by GLM model**

| variable | permutation | dropout_loss | label |
| --- | --- | --- | --- |
| CXCL1 | 0 | 0.570847921134723 | GLM |
| VIM | 0 | 0.577787237523939 | GLM |
| CPQ | 0 | 0.584491678325392 | GLM |
| VCAN | 0 | 0.595095928184249 | GLM |
| CD3D | 0 | 0.61413469299591 | GLM |

**Table 7 Important genes by RF model**

| variable | permutation | dropout_loss | label |
| --- | --- | --- | --- |
| ECSCR | 0 | 0.309028756562737 | RF |
| CXCL12 | 0 | 0.309514853726171 | RF |
| KDR | 0 | 0.311290458004191 | RF |
| CXCL1 | 0 | 0.316134134078013 | RF |
| CLN8 | 0 | 0.318239873335846 | RF |

**Table 8 Important genes by SVM model**

| variable | permutation | dropout_loss | label |
| --- | --- | --- | --- |
| RGS1 | 0 | 0.288490702663185 | SVM |
| ACAT2 | 0 | 0.288529340295523 | SVM |
| KDR | 0 | 0.288807162451411 | SVM |
| TUBB2A | 0 | 0.288938766453413 | SVM |
| TDO2 | 0 | 0.290361646803278 | SVM |

**Table 9 Important genes by XGB model**

| variable | permutation | dropout_loss | label |
| --- | --- | --- | --- |
| CLN8 | 0 | 0.326344683962264 | XGB |
| CXCL12 | 0 | 0.327758893125846 | XGB |
| TDO2 | 0 | 0.328013535341694 | XGB |
| FPR1 | 0 | 0.328088168478638 | XGB |
| SERPINB9 | 0 | 0.331438300477322 | XGB |
