# Supplementary material for: Vitamin A family suppresses periodontitis by restoring mitochondrial metabolic reprogramming in macrophages through JAK-STAT pathway
Source: Front Genet. 2025 Jan 28;16:1505933. doi: 10.3389/fgene.2025.1505933 (PMC11810908; doi:10.3389/fgene.2025.1505933)
Supplement: Supplementary file 2 [file Table2.docx]

https://www.jianguoyun.com/p/DbDLOQYQ-Mz8DBiAhOYFIAA
